# Supplementary material for: Complications of Robotic Video-Assisted Thoracoscopic Surgery Compared to Open Thoracotomy for Resectable Non-Small Cell Lung Cancer
Source: J Pers Med. 2022 Aug 12;12(8):1311. doi: 10.3390/jpm12081311 (PMC9410342; doi:10.3390/jpm12081311)
Supplement: Supplementary file 1 [file jpm-12-01311-s001.zip › jpm-1832092-supplementary.pdf]

Journal of *Personalized Medicine*

MDPI

Supplementary Materials

Study or Subgroup	RVATS Mean	RVATS SD	RVATS Total	Open Mean	Open SD	Open Total	Weight	Std. Mean Difference IV, Random, 95% CI	Year
Cerfolio 2011	132	60	106	90	48	318	7.4%	0.82 [0.59, 1.04]	2011
Adams 2014	242	65	120	176	84	5913	7.6%	0.79 [0.61, 0.97]	2014
Farivar 2014	199	58	181	244	88	5913	7.7%	−0.52 [−0.66, −0.37]	2014
Oh 2017	276	95	2775	235	94	2775	7.8%	0.43 [0.38, 0.49]	2017
Gallagher 2018	209	51	100	197	37	57	6.9%	0.26 [−0.07, 0.58]	2018
Gu 2018	155	45	17	150	48	86	5.9%	0.10 [−0.42, 0.62]	2018
Novellis 2018	155	37	23	122	33	38	5.7%	0.94 [0.40, 1.49]	2018
Kneuertz 2019	287	13	296	279	21	240	7.6%	0.47 [0.30, 0.64]	2019
Nelson 2019	222	58	106	149	44	424	7.4%	1.55 [1.31, 1.78]	2019
Qiu 2020	200	73	49	240	48	66	6.7%	−0.66 [−1.04, −0.28]	2020
Huang 2021	104	41	76	102	29	72	7.0%	0.06 [−0.27, 0.38]	2021
Kent 2021	160	80	500	164	58	480	7.7%	−0.06 [−0.18, 0.07]	2021
Zhou 2022	212	73	77	145	61	105	7.0%	1.01 [0.69, 1.32]	2022
Kneuertz 2022	239	92	634	227	80	562	7.7%	0.14 [0.02, 0.25]	2022
**Total (95% CI)**			**5060**			**17049**	**100.0%**	**0.38 [0.13, 0.63]**	

Heterogeneity: $Tau^2 = 0.20$; $Chi^2 = 390.01$, df = 13 ($P < 0.00001$); $I^2 = 97\%$
Test for overall effect: Z = 2.98 (P = 0.003)

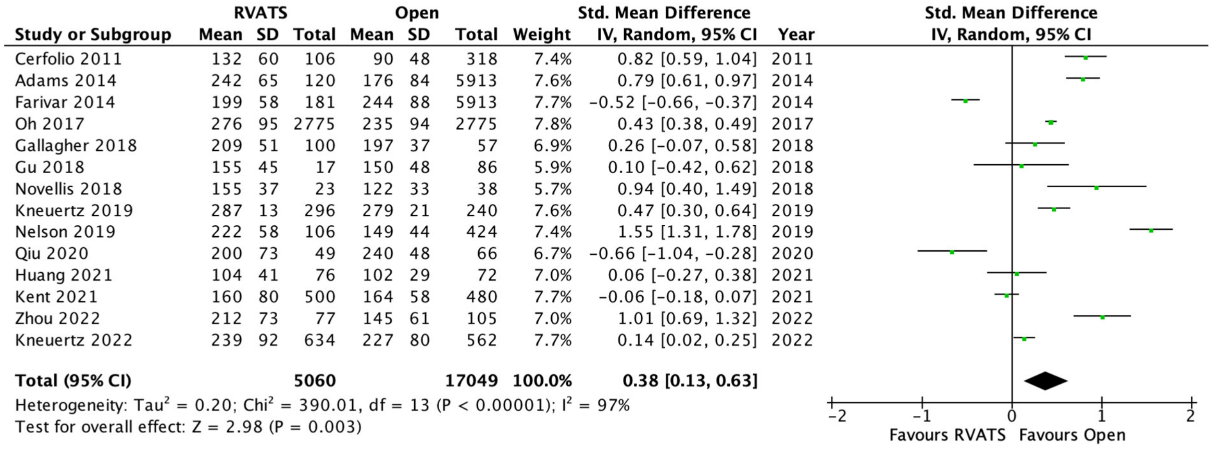

Figure S1. Operation time in patients who underwent robotic video-assisted thoracoscopic surgery compared with open thoracotomy for resectable non-small cell lung cancer.

Study or Subgroup	RVATS Mean	RVATS SD	RVATS Total	Open Mean	Open SD	Open Total	Weight	Std. Mean Difference IV, Random, 95% CI	Year
Cerfolio 2011	3	1	106	19	11	318	5.9%	−1.67 [−1.92, −1.43]	2011
Adams 2014	5	3	120	7	8	5913	6.1%	−0.25 [−0.43, −0.07]	2014
Farivar 2014	3	3	181	7	8	5913	6.2%	−0.51 [−0.65, −0.36]	2014
Kwon 2017	16	11	74	21	13	201	5.8%	−0.40 [−0.67, −0.13]	2017
Oh 2017	7	6	2775	9	6	2775	6.4%	−0.33 [−0.39, −0.28]	2017
Rajaram 2017	6	6	3689	7	7	45527	6.4%	−0.14 [−0.18, −0.11]	2017
Yang 2017	10	6	172	10	5	157	6.0%	0.00 [−0.22, 0.22]	2017
Gallagher 2018	9	4	100	13	5	57	5.5%	−0.91 [−1.25, −0.57]	2018
Gu 2018	11	8	17	10	4	86	4.6%	0.20 [−0.32, 0.73]	2018
Novellis 2018	4	2	23	8	7	38	4.5%	−0.70 [−1.23, −0.16]	2018
Kneuertz 2019	4	2	296	6	3	240	6.1%	−0.80 [−0.98, −0.62]	2019
Nelson 2019	4	2	106	5	1	424	6.0%	−0.79 [−1.01, −0.57]	2019
Subramania 2019	4	2	1929	7	3	8501	6.4%	−1.06 [−1.11, −1.00]	2019
Huang 2021	10	4	76	12	4	72	5.6%	−0.50 [−0.82, −0.17]	2021
Kent 2021	4	5	885	6	5	885	6.4%	−0.40 [−0.49, −0.31]	2021
Kneuertz 2022	4	4	634	5	4	562	6.3%	−0.25 [−0.36, −0.14]	2022
Zhou 2022	3	2	77	4	2	105	5.7%	−0.50 [−0.80, −0.20]	2022
**Total (95% CI)**			**11260**			**71774**	**100.0%**	**−0.53 [−0.74, −0.32]**	

Heterogeneity: $Tau^2 = 0.18$; $Chi^2 = 1025.09$, df = 16 ($P < 0.00001$); $I^2 = 98\%$
Test for overall effect: Z = 5.01 (P < 0.00001)

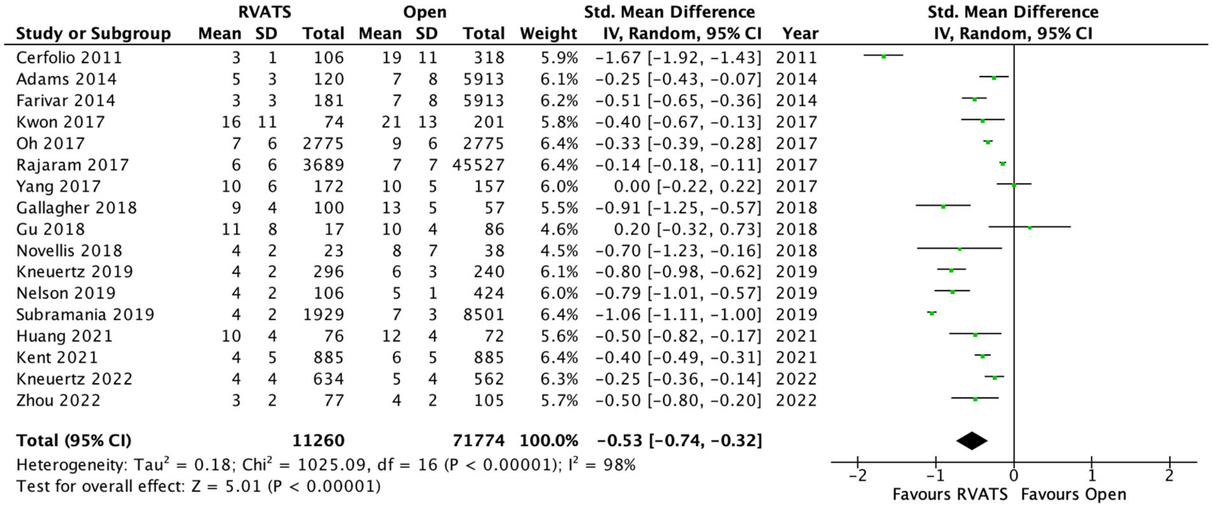

Figure S2. Length of stay in patients who underwent robotic video-assisted thoracoscopic surgery compared with open thoracotomy for resectable non-small cell lung cancer.

Study or Subgroup	RVATS Mean	RVATS SD	RVATS Total	Open Mean	Open SD	Open Total	Weight	Std. Mean Difference IV, Random, 95% CI	Year
Cerfolio 2011	3	1	106	19	11	318	11.4%	−1.67 [−1.92, −1.43]	2011
Adams 2014	3	4	120	5	4	5913	11.8%	−0.50 [−0.68, −0.32]	2014
Farivar 2014	3	3	181	5	4	5913	11.9%	−0.50 [−0.65, −0.36]	2014
Kwon 2017	14	10	74	13	7	201	11.2%	0.13 [−0.14, 0.39]	2017
Gallagher 2018	7	4	100	10	5	57	10.7%	−0.68 [−1.01, −0.35]	2018
Gu 2018	9	9	17	7	3	86	9.1%	0.44 [−0.08, 0.96]	2018
Huang 2021	4	1	76	5	2	72	10.8%	−0.63 [−0.96, −0.30]	2021
Kent 2021	4	5	885	5	5	885	12.1%	−0.20 [−0.29, −0.11]	2021
Zhou 2022	2	2	77	3	2	105	11.0%	−0.50 [−0.80, −0.20]	2022
**Total (95% CI)**			**1636**			**13550**	**100.0%**	**−0.48 [−0.78, −0.17]**	

Heterogeneity: $Tau^2 = 0.20$; $Chi^2 = 152.36$, df = 8 ($P < 0.00001$); $I^2 = 95\%$
Test for overall effect: Z = 3.03 (P = 0.002)

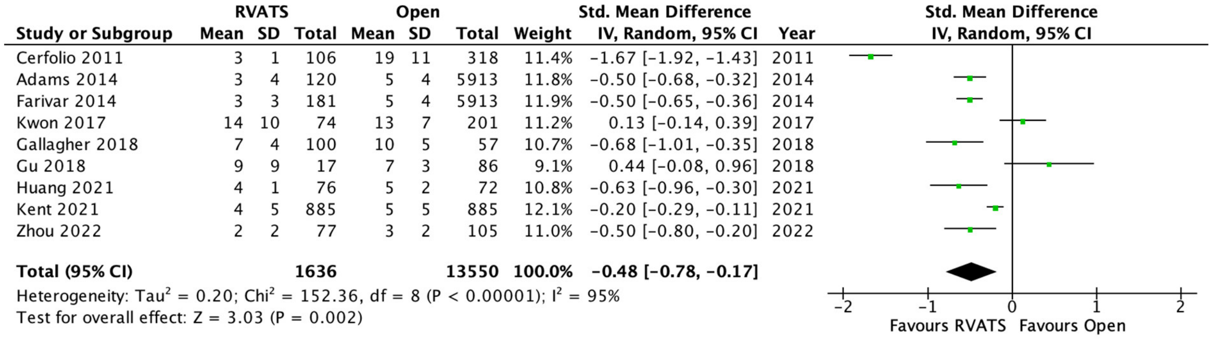

Figure S3. Chest drain duration in patients who underwent robotic video-assisted thoracoscopic surgery compared with open thoracotomy for resectable non-small cell lung cancer.

Study or Subgroup	RVATS Mean	RVATS SD	RVATS Total	Open Mean	Open SD	Open Total	Weight	Std. Mean Difference IV, Random, 95% CI	Year
Novellis 2018	5	1	23	4	1	38	10.4%	0.99 [0.44, 1.54]	2018
Gallagher 2018	5	2	100	4	1	57	18.0%	0.58 [0.25, 0.91]	2018
Nelson 2019	6	2	106	5	1	424	23.8%	0.79 [0.57, 1.01]	2019
Zhou 2022	6	2	77	4	2	105	19.0%	1.00 [0.68, 1.31]	2022
Kneuertz 2022	5	2	634	4	2	562	28.8%	0.50 [0.38, 0.61]	2022
**Total (95% CI)**			**940**			**1186**	**100.0%**	**0.73 [0.51, 0.95]**	

Heterogeneity: $Tau^2 = 0.04$; $Chi^2 = 13.86$, df = 4 (P = 0.008); $I^2 = 71\%$
Test for overall effect: Z = 6.60 (P < 0.00001)

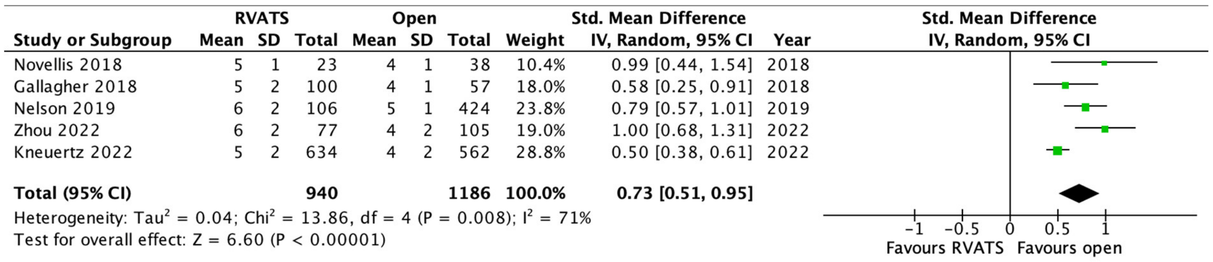

Figure S4. Stations harvested in patients who underwent robotic video-assisted thoracoscopic surgery compared with open thoracotomy for resectable non-small cell lung cancer.

Study or Subgroup	RVATS Events	RVATS Total	Open Events	Open Total	Weight	Odds Ratio IV, Random, 95% CI	Year
Veronesi 2010	0	54	3	54	3.3%	0.13 [0.01, 2.68]	
Adams 2014	1	120	461	5913	6.3%	0.10 [0.01, 0.71]	2014
Farivar 2014	0	181	461	5913	3.7%	0.03 [0.00, 0.52]	2014
Oh 2017	105	2775	150	2775	21.3%	0.69 [0.53, 0.89]	2017
Gu 2018	2	17	5	86	7.5%	2.16 [0.38, 12.18]	2018
Kneuertz 2019	12	296	24	240	16.7%	0.38 [0.19, 0.78]	2019
Kent 2021	13	885	67	885	18.0%	0.18 [0.10, 0.33]	2021
Kneuertz 2022	17	634	25	562	17.7%	0.59 [0.32, 1.11]	2022
Zhou 2022	1	77	6	105	5.6%	0.22 [0.03, 1.84]	2022
**Total (95% CI)**		**5039**		**16533**	**100.0%**	**0.37 [0.20, 0.66]**	
Total events	151		1202				

Heterogeneity: $Tau^2 = 0.40$; $Chi^2 = 27.75$, df = 8 (P = 0.0005); $I^2 = 71\%$
Test for overall effect: Z = 3.37 (P = 0.0007)

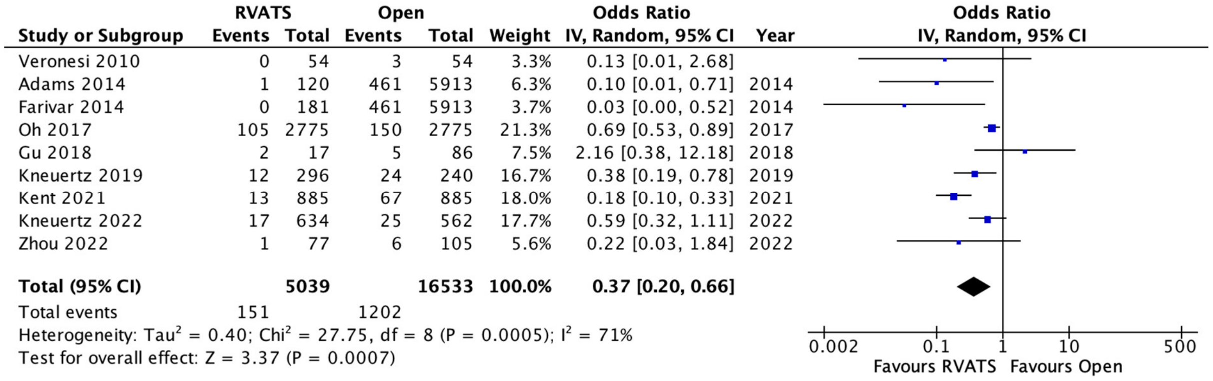

Figure S5. Postoperative transfusion in patients who underwent robotic video-assisted thoracoscopic surgery compared with open thoracotomy for resectable non-small cell lung cancer.

Study or Subgroup	RVATS Events	RVATS Total	Open Events	Open Total	Weight	Odds Ratio IV, Random, 95% CI	Year
Adams 2014	2	120	302	5913	3.6%	0.31 [0.08, 1.28]	2014
Farivar 2014	3	181	302	5913	5.1%	0.31 [0.10, 0.99]	2014
Yang 2017	5	172	8	157	5.2%	0.56 [0.18, 1.74]	2017
Gallagher 2018	12	100	8	57	6.9%	0.84 [0.32, 2.18]	2018
Gu 2018	2	17	8	86	2.7%	1.30 [0.25, 6.74]	2018
Nelson 2019	7	106	17	424	7.5%	1.69 [0.68, 4.19]	2019
Subramania 2019	100	1929	859	8501	29.2%	0.49 [0.39, 0.60]	2019
Kneuertz 2019	9	296	19	240	8.9%	0.36 [0.16, 0.82]	2019
Qiu 2020	2	49	3	66	2.2%	0.89 [0.14, 5.56]	2020
Huang 2021	3	76	6	72	3.5%	0.45 [0.11, 1.88]	2021
Nawalanie 2022	16	211	25	210	11.9%	0.61 [0.31, 1.17]	2022
Kneuertz 2022	23	634	21	562	13.4%	0.97 [0.53, 1.77]	2022
**Total (95% CI)**		**3891**		**22201**	**100.0%**	**0.61 [0.46, 0.81]**	
Total events	184		1578				

Heterogeneity: $Tau^2 = 0.06$; $Chi^2 = 15.30$, df = 11 (P = 0.17); $I^2 = 28\%$
Test for overall effect: Z = 3.43 (P = 0.0006)

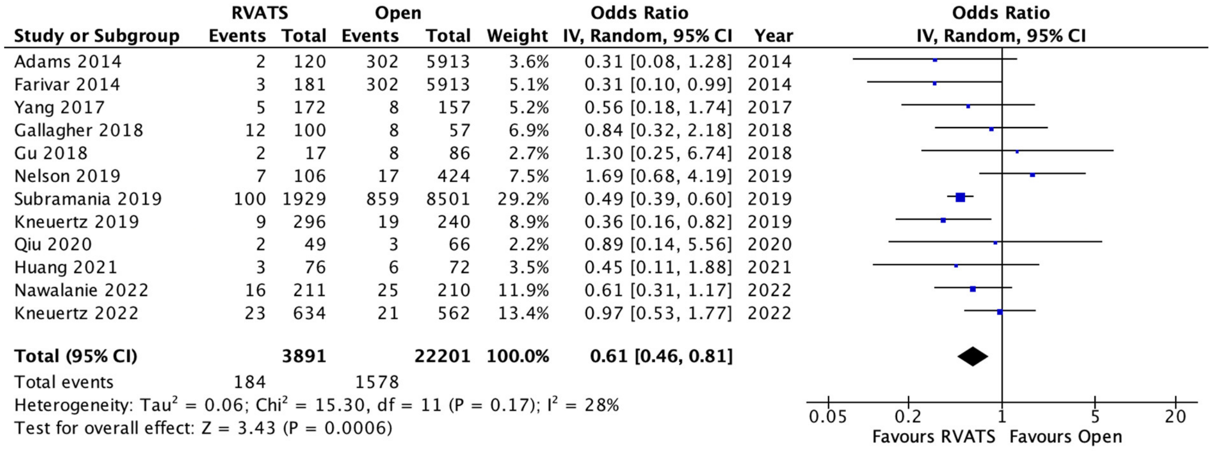

Figure S6. Pneumonia in patients who underwent robotic video-assisted thoracoscopic surgery compared with open thoracotomy for resectable non-small cell lung cancer.

Study or Subgroup	RVATS Events	RVATS Total	Open Events	Open Total	Weight	Odds Ratio IV, Random, 95% CI	Year
Adams 2014	6	120	639	5913	7.5%	0.43 [0.19, 0.99]	2014
Deen 2014	0	57	0	69		Not estimable	2014
Farivar 2014	11	181	633	5913	8.8%	0.54 [0.29, 1.00]	2014
Oh 2017	280	2775	250	2775	11.1%	1.13 [0.95, 1.36]	2017
Yang 2017	15	172	7	157	6.9%	2.05 [0.81, 5.16]	2017
Kwon 2017	2	74	20	201	4.3%	0.25 [0.06, 1.10]	2017
Nelson 2019	16	106	68	424	9.0%	0.93 [0.52, 1.68]	2019
Subramania 2019	154	1929	323	8501	11.1%	2.20 [1.80, 2.68]	2019
Kneuertz 2019	15	296	22	240	8.4%	0.53 [0.27, 1.04]	2019
Qiu 2020	3	49	2	66	3.2%	2.09 [0.34, 13.00]	2020
Huang 2021	6	76	7	72	5.7%	0.80 [0.25, 2.49]	2021
Nawalanie 2022	16	211	43	210	8.9%	0.32 [0.17, 0.59]	2022
Zhou 2022	3	77	14	105	5.0%	0.26 [0.07, 0.95]	2022
Kneuertz 2022	51	634	39	562	10.0%	1.17 [0.76, 1.81]	2022
**Total (95% CI)**		**6757**		**25208**	**100.0%**	**0.80 [0.54, 1.17]**	
Total events	578		2067				

Heterogeneity: $Tau^2 = 0.34$; $Chi^2 = 84.16$, df = 12 ($P < 0.00001$); $I^2 = 86\%$
Test for overall effect: Z = 1.16 (P = 0.25)

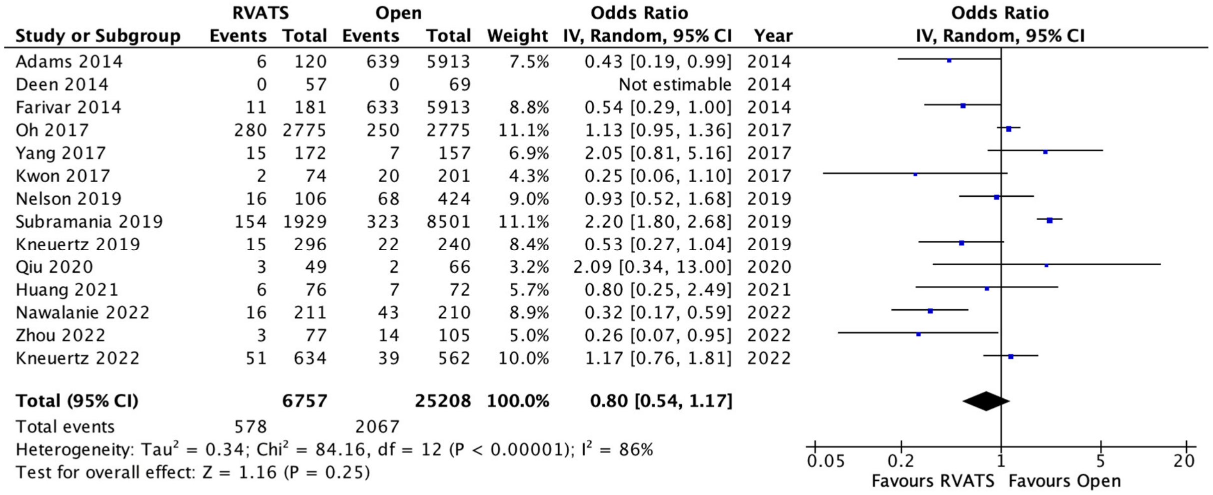

Figure S7. Prolonged air leak in patients who underwent robotic video-assisted thoracoscopic surgery compared with open thoracotomy for resectable non-small cell lung cancer.

Study or Subgroup	RVATS Events	RVATS Total	Open Events	Open Total	Weight	Odds Ratio IV, Random, 95% CI	Year
Farivar 2014	3	181	313	5913	8.7%	0.30 [0.10, 0.95]	2014
Oh 2017	344	2775	436	2775	30.0%	0.76 [0.65, 0.88]	2017
Yang 2017	5	172	4	157	6.9%	1.15 [0.30, 4.34]	2017
Kneuertz 2019	15	296	38	240	17.7%	0.28 [0.15, 0.53]	2019
Qiu 2020	2	49	4	66	4.5%	0.66 [0.12, 3.75]	2020
Kneuertz 2022	16	634	17	562	16.1%	0.83 [0.42, 1.66]	2022
Nawalanie 2022	13	211	27	210	16.1%	0.45 [0.22, 0.89]	2022
**Total (95% CI)**		**4318**		**9923**	**100.0%**	**0.56 [0.38, 0.83]**	
Total events	398		839				

Heterogeneity: $Tau^2 = 0.13$; $Chi^2 = 13.60$, df = 6 ($P = 0.03$); $I^2 = 56\%$
Test for overall effect: Z = 2.86 (P = 0.004)

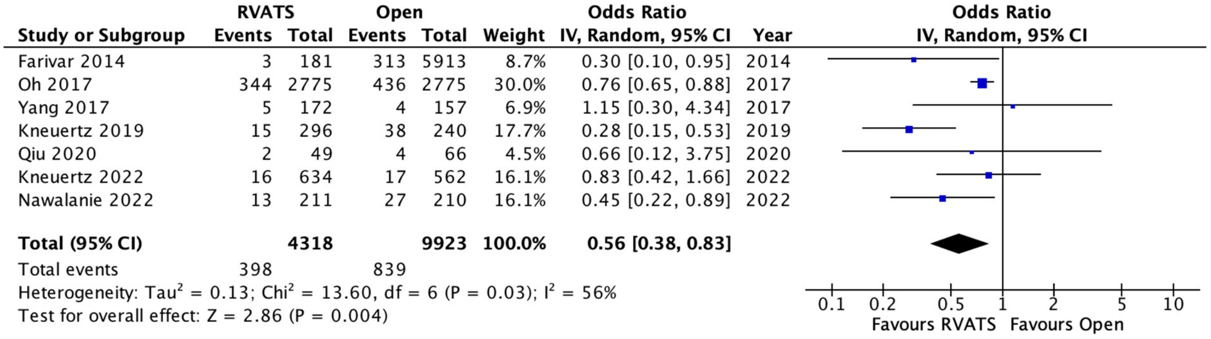

Figure S8. Atelectasis in patients who underwent robotic video-assisted thoracoscopic surgery compared with open thoracotomy for resectable non-small cell lung cancer.

Study or Subgroup	RVATS Events	RVATS Total	Open Events	Open Total	Weight	Odds Ratio IV, Random, 95% CI	Year
Deen 2014	0	57	0	69		Not estimable	2014
Farivar 2014	0	181	35	5913	2.8%	0.46 [0.03, 7.47]	2014
Oh 2017	8	2775	11	2775	26.1%	0.73 [0.29, 1.81]	2017
Yang 2017	1	172	1	157	2.8%	0.91 [0.06, 14.71]	2017
Subramania 2019	14	1929	73	8501	65.9%	0.84 [0.48, 1.50]	2019
Qiu 2020	0	49	0	66		Not estimable	2020
Kneuertz 2022	3	634	0	562	2.5%	6.24 [0.32, 120.97]	2022
**Total (95% CI)**		**5797**		**18043**	**100.0%**	**0.84 [0.53, 1.34]**	
Total events	26		120				

Heterogeneity: $Tau^2 = 0.00$; $Chi^2 = 2.04$, df = 4 ($P = 0.73$); $I^2 = 0\%$
Test for overall effect: Z = 0.73 (P = 0.46)

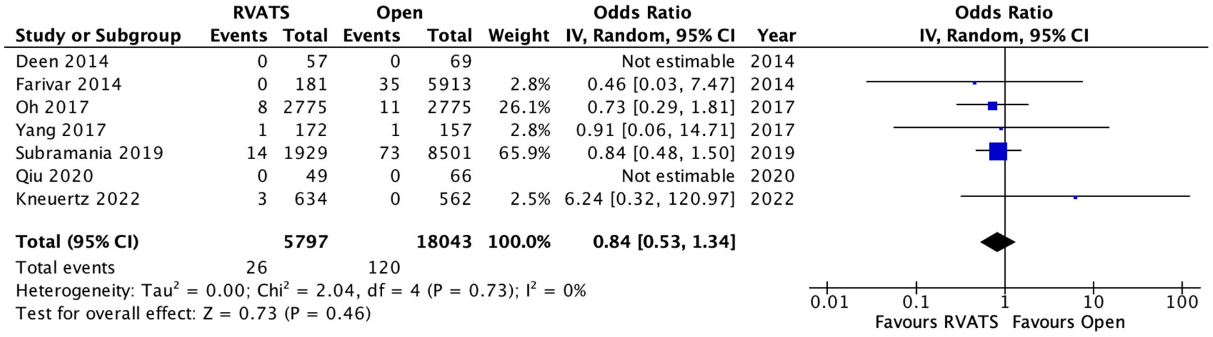

Figure S9. Myocardial infarction in patients who underwent robotic video-assisted thoracoscopic surgery compared with open thoracotomy for resectable non-small cell lung cancer.

Study or Subgroup	RVATS Events	RVATS Total	Open Events	Open Total	Weight	Odds Ratio IV, Random, 95% CI	Year
Farivar 2014	0	181	41	5913	6.4%	0.39 [0.02, 6.36]	2014
Oh 2017	28	2775	44	2775	43.6%	0.63 [0.39, 1.02]	2017
Yang 2017	3	172	2	157	13.1%	1.38 [0.23, 8.34]	2017
Subramania 2019	0	1929	111	8501	6.4%	0.02 [0.00, 0.31]	2019
Huang 2021	1	76	0	72	4.9%	2.88 [0.12, 71.87]	2021
Kneuertz 2022	6	634	8	562	25.6%	0.66 [0.23, 1.92]	2022
**Total (95% CI)**		**5767**		**17980**	**100.0%**	**0.59 [0.28, 1.26]**	
Total events	38		206				

Heterogeneity: Tau^2 = 0.28; Chi^2 = 7.71, df = 5 (P = 0.17); I^2 = 35%
Test for overall effect: Z = 1.37 (P = 0.17)

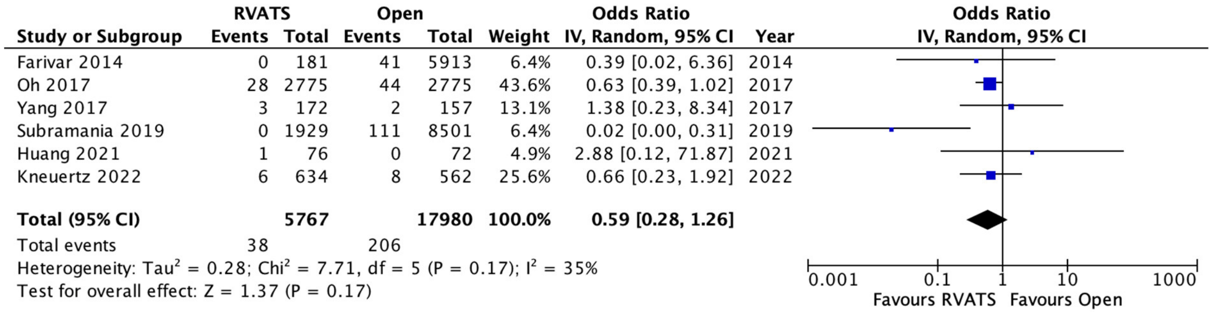

Figure S10. Thromboembolism in patients who underwent robotic video-assisted thoracoscopic surgery compared with open thoracotomy for resectable non-small cell lung cancer.

Study or Subgroup	RVATS Events	RVATS Total	Open Events	Open Total	Weight	Odds Ratio IV, Random, 95% CI	Year
Adams 2014	7	120	260	5913	17.3%	1.35 [0.62, 2.92]	2014
Farivar 2014	1	181	65	5913	4.1%	0.50 [0.07, 3.62]	2014
Nelson 2019	2	106	13	424	6.6%	0.61 [0.14, 2.74]	2019
Kneuertz 2019	3	296	10	240	8.4%	0.24 [0.06, 0.87]	2019
Kent 2021	26	885	43	885	26.3%	0.59 [0.36, 0.97]	2021
Kneuertz 2022	19	634	12	562	18.4%	1.42 [0.68, 2.94]	2022
Nawalanie 2022	11	211	29	562	18.9%	1.01 [0.50, 2.06]	2022
**Total (95% CI)**		**2433**		**14499**	**100.0%**	**0.82 [0.54, 1.25]**	
Total events	69		432				

Heterogeneity: Tau^2 = 0.11; Chi^2 = 9.62, df = 6 (P = 0.14); I^2 = 38%
Test for overall effect: Z = 0.94 (P = 0.35)

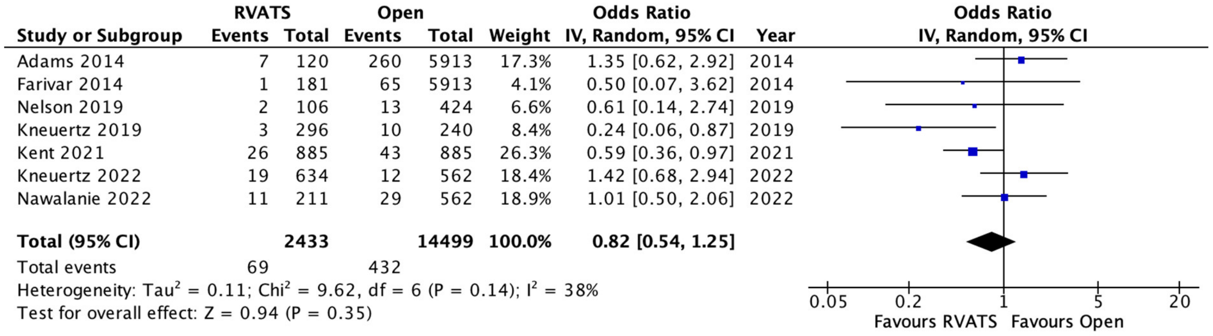

Figure S11. Reoperation in patients who underwent robotic video-assisted thoracoscopic surgery compared with open thoracotomy for resectable non-small cell lung cancer.

Study or Subgroup	RVATS Events	RVATS Total	Open Events	Open Total	Weight	Odds Ratio IV, Random, 95% CI	Year
Oh 2017	19	2775	47	2775	33.1%	0.40 [0.23, 0.68]	2017
Yang 2017	3	172	0	157	4.3%	6.50 [0.33, 126.93]	2017
Subramania 2019	11	1929	54	8501	29.9%	0.90 [0.47, 1.72]	2019
Kneuertz 2019	9	296	5	240	19.0%	1.47 [0.49, 4.46]	2019
Kent 2021	1	885	2	885	6.2%	0.50 [0.05, 5.52]	2021
Zhou 2022	1	77	6	105	7.6%	0.22 [0.03, 1.84]	2022
**Total (95% CI)**		**6134**		**12663**	**100.0%**	**0.71 [0.37, 1.36]**	
Total events	44		114				

Heterogeneity: Tau^2 = 0.26; Chi^2 = 9.52, df = 5 (P = 0.09); I^2 = 47%
Test for overall effect: Z = 1.03 (P = 0.30)

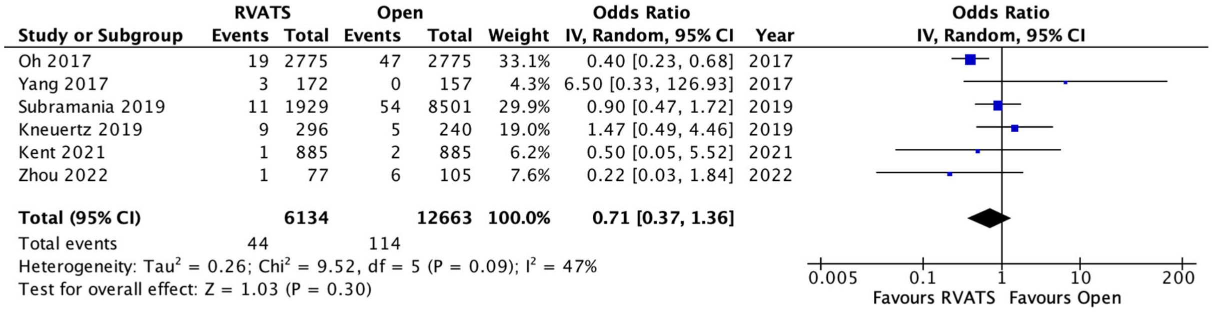

Figure S12. Wound infection in patients who underwent robotic video-assisted thoracoscopic surgery compared with open thoracotomy for resectable non-small cell lung cancer.
